# Mob4-dependent STRIPAK involves the chaperonin TRiC to coordinate myofibril and microtubule network growth

**DOI:** 10.1371/journal.pgen.1010287

**Published:** 2022-06-23

**Authors:** Joachim Berger, Silke Berger, Peter D. Currie

**Affiliations:** 1 Australian Regenerative Medicine Institute, Monash University, Clayton, Australia; 2 Victoria Node, EMBL Australia, Clayton, Australia; The University of Maine, UNITED STATES

## Abstract

Myofibrils of the skeletal muscle are comprised of sarcomeres that generate force by contraction when myosin-rich thick filaments slide past actin-based thin filaments. Surprisingly little is known about the molecular processes that guide sarcomere assembly *in vivo*, despite deficits within this process being a major cause of human disease. To overcome this knowledge gap, we undertook a forward genetic screen coupled with reverse genetics to identify genes required for vertebrate sarcomere assembly. In this screen, we identified a zebrafish mutant with a nonsense mutation in *mob4*. In *Drosophila*, *mob4* has been reported to play a role in spindle focusing as well as neurite branching and in planarians *mob4* was implemented in body size regulation. In contrast, zebrafish *mob4*^*geh*^ mutants are characterised by an impaired actin biogenesis resulting in sarcomere defects. Whereas loss of *mob4* leads to a reduction in the amount of myofibril, transgenic expression of *mob4* triggers an increase. Further genetic analysis revealed the interaction of Mob4 with the actin-folding chaperonin TRiC, suggesting that Mob4 impacts on TRiC to control actin biogenesis and thus myofibril growth. Additionally, *mob4*^*geh*^ features a defective microtubule network, which is in-line with tubulin being the second main folding substrate of TRiC. We also detected similar characteristics for *strn3*-deficient mutants, which confirmed Mob4 as a core component of STRIPAK and surprisingly implicates a role of the STRIPAK complex in sarcomerogenesis.

## Author summary

Within muscle, highly organised filaments slide over each other to generate the force required for the movement of our bodies. Assembly of this contractile apparatus is not well understood and its regulation remains enigmatic.

To gain novel insights into these processes we facilitated a zebrafish screen that resulted in the discovery of a novel molecule involved in the coordination of the contractile apparatus assembly: The highly conserved MOB family member Mob4. Whereas loss of mob4 led to impaired actin biogenesis and defects in the contractile apparatus assembly, which resembled aspects of human myopathy disorders, gain of mob4 function resulted in a higher amount of contractile apparatus. Further analyses of strn3-deficient mutants demonstrated that Mob4 functions within a protein complex called striatin-interacting phosphatases and kinases (STRIPAK). Mob4 also involves another protein complex called TRiC, which is required for actin and tubulin biogenesis. Whereas actin is the main component of the muscle’s thin filaments, tubulin constitutes the microtubule network essential for neuronal axons. Accordingly, all analysed mutants for mob4 and strn3 featured neuronal as well as muscle defects. We thus conclude that the two protein complexes STRIPAK and TRiC interact through Mob4 to coordinate growth of the myofibril and microtubule network.

## Introduction

Skeletal muscle has a remarkable plasticity, as it can regenerate even repeated traumas and rapidly shrinks during physical inactivity or aging (sarcopenia), making muscle weakness a major contributor to both mortality and morbidity [1]. Likewise, deficits in sarcomere assembly lead to impaired myofibril function, resulting in heterogeneous muscle diseases including myopathies [2]. However, little is known about the molecular pathways that regulate these processes and how the muscle’s sarcomeres are assembled [3].

The functional units of the myofibril are the highly ordered sarcomeres, which are mainly comprised of interdigitating myosin-rich thick and actin-based thin filaments. A comprehensive description of sarcomere assembly has not been fully established, but different theories have been brought forward. These include the model describing independent I-Z-I complexes that recruit titin to form myofibril [4,5] and the premyofibril model that elucidates the maturation of Z-body-containing premyofibril into myofibril [6]. It is also established that mechanical tension is required to trigger myofibril assembly [7]. Likewise, the details of the assembly of sarcomeric thin filaments are still enigmatic. Current models suggest that this process initiates at the Z-disc of sarcomeres, where monomeric α-actin is folded by the chaperonin complex TRiC (T-complex polypeptide-1 ring complex or CCT) [8]. Folded actin is passed on to the co-chaperon Bag3 that interacts with the capping protein CapZ and the Z-disc protein α-actinin to initiate actin polymerisation along the nebulin scaffold [9–13]. Subsequent polymerisation dynamics and final capping of thin filaments is mediated by Leiomodins and Tropomodulin4 [14,15]. In addition to skeletal muscle α-actin, TRiC also folds α- and β-tubulin that polymerise to form microtubules required also for neurite formation [16]. Accordingly, TRiC loss-of-function in zebrafish is characterised by neuronal as well as sarcomeric defects provoked by a compromised microtubule and thin filament assembly, respectively [8].

Another macromolecular complex is the striatin-interacting phosphatases and kinases (STRIPAK) complex that interacts with a number of different signalling pathways, resulting in numerous cellular and developmental roles for STRIPAK [17]. Whereas many isoforms and paralogs of STRIPAK subunits can assemble into various STRIPAK variants, Mob4 is a core protein of STRIPAK. Also known as phocein, Mob4 belongs to the family of MOBs (monopolar spindle-one-binder proteins) that are highly conserved in eukaryotes [18]. In *Drosophila*, Mob4 has been reported to play a role in the focusing of microtubule-based spindles as well as in axonal microtubule organization and associated neurite branching [19,20]. As demonstrated in rats, the neurological functions of *mob4* depend on the STRIPAK complex [21]. However, various additional roles have been attributed to Mob4, including the limitation of differentiating WNT-signalling midline muscle cells to regulate the body size of planarians [22], coordination of Hippo and insulin-like receptor signalling to reactivate neural stem cells [23], or regulation of Hippo to control proliferation of pancreatic cancer cells [24]. Interestingly, Dlg5 and Slmap within STRIPAK regulate expression of sarcomeric genes via the Hippo pathway [25], which also regulates protein synthesis in mouse muscle [26].

To discover novel molecules involved in thin filament assembly and better understand this process, we initiated a genetic screen in zebrafish that resulted in the identification of *mob4*-deficient mutants with sarcomeric defects and aggregates that resembled aspects of human nemaline myopathy. We reveal that the interaction of Mob4 with TRiC is required for the coordination of sarcomere assembly. Comparable to TRiC deficiency, the microtubule network of retinal ganglion cells is compromised in *mob4*^*geh*^ mutants. Additionally, loss of the STRIPAK scaffold Strn3 within generated zebrafish mutants resemble the defects found in *mob4*- and TRiC-deficient mutants. Thus, the Mob4 component of STRIPAK might regulate TRiC function to coordinate growth of the myofibril and the microtubule network.

## Results

### Muscle integrity is compromised in the zebrafish mutant *gemütlich*

**Fig 1:**
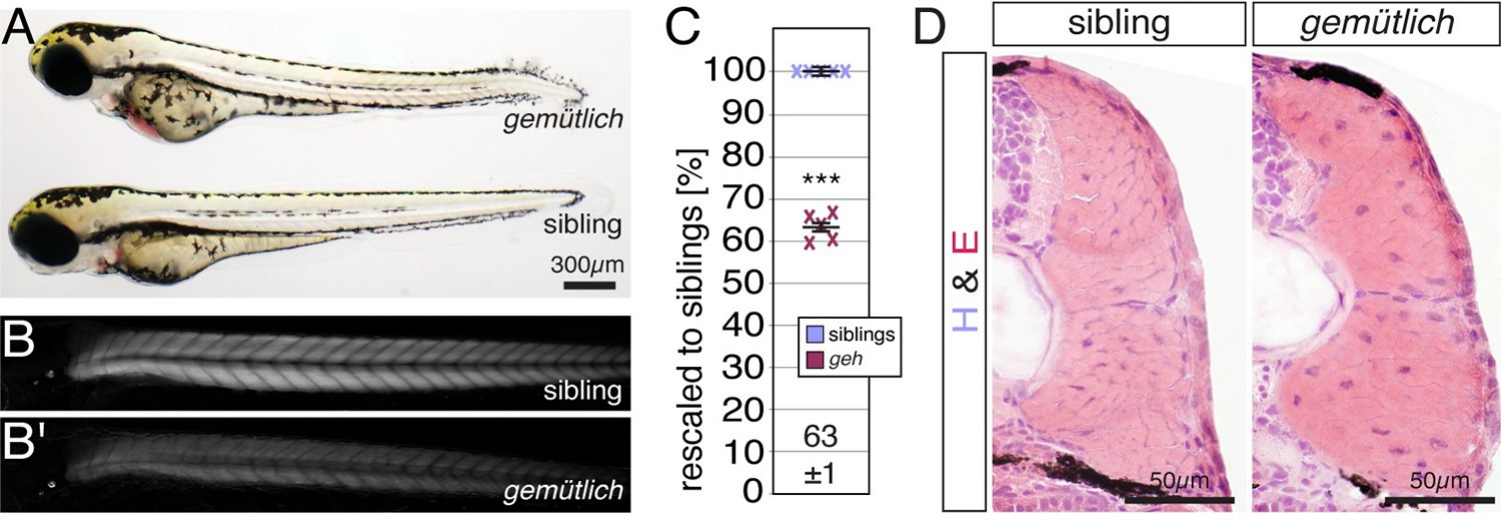
The muscle integrity is compromised in *gemütlich* (*geh*) mutants. (A) At 3 dpf, *geh* mutants appeared unremarkable under bright-field microscopy. (B) In comparison to their siblings, (B’) 3-dpf-old *geh* homozygotes appeared darker in representative images taken under polarised light conditions. (C) After rescaling to siblings (100 ± 1%), the birefringence of 3-dpf-old *geh* homozygotes was significantly reduced to 61 ± 1%. Crosses represent averaged birefringence of clutches with a minimum of 6 larvae per genotype (n = 5 clutches). Data are presented as mean ± SEM; *** P < 0.001 calculated by Student’s t-test. (D) Fibrotic signs were not detected on H&E-stained cross sections of 3-dpf-old *geh* homozygotes and siblings (n = 6 per genotype). Scale bar sizes are indicated.

To study sarcomere assembly, a forward genetic screen was performed in zebrafish utilising muscle birefringence. Birefringence is a feature of the pseudo-crystalline myofibril that enables muscle fibres to appear bright under polarized light in an otherwise dark environment. Thus, myofibril defects can be readily detected under polarized light and quantified by measuring the muscle’s brightness [27]. Mutations were randomly introduced by N-ethyl-N-nitrosourea in male zebrafish and germline mutations were stabilised by out-crossing of the males over two generations [28]. 126 F2 families were established and screened for myofibril deficiencies by birefringence analysis at 3 days post fertilization (dpf). One zebrafish mutant that appeared unremarkable under brightfield conditions showed a birefringence reduction under polarised light (Fig 1A and 1B). Quantification of the birefringence at 3 dpf revealed that the birefringence of this mutant was significantly reduced compared to the siblings, indicating that the amount of organised myofibril could be diminished (Fig 1C). Accordingly, although the mutant was touch sensitive at 3 dpf (S1 Video), its motility and forward thrust was impaired compared to the siblings (S2 Video). In relation to the muscle phenotype, this mutant was named *gemütlich* (*geh*), German for laid-back. In contrast to viable siblings and starved siblings that died at 11 dpf, *geh* homozygotes progressively showed cardiac edema and signs of impaired movement and did not survive past 6 dpf, indicating that *geh* mutants potentially harbour defects in addition to a muscle-related inability to hunt for food. To further assess the muscle of *geh* mutants, cross sections were H&E-stained at 3 dpf. Neither signs of fibrosis nor dystrophic fibers were detected on H&E-stained sections of *geh* homozygotes, indicating that degradation of entire myofibres, typically seen in dystrophic muscle [29], are absent (Fig 1D). Cross-sectional areas (CSA) of *geh* homozygous skeletal muscle (0.028 ± 0.001 mm^2^) were comparable to siblings (0.0280 ± 0.0008 mm^2^) (n = 5, P = 0.95 calculated by Student’s t-test).

Taken together, a genetic screen resulted in the isolation of the zebrafish mutant *gemütlich* that feature a significant reduction in birefringence.

### The zebrafish mutant *gemütlich* harbours a nonsense mutation in *mob4*

**Fig 2:**
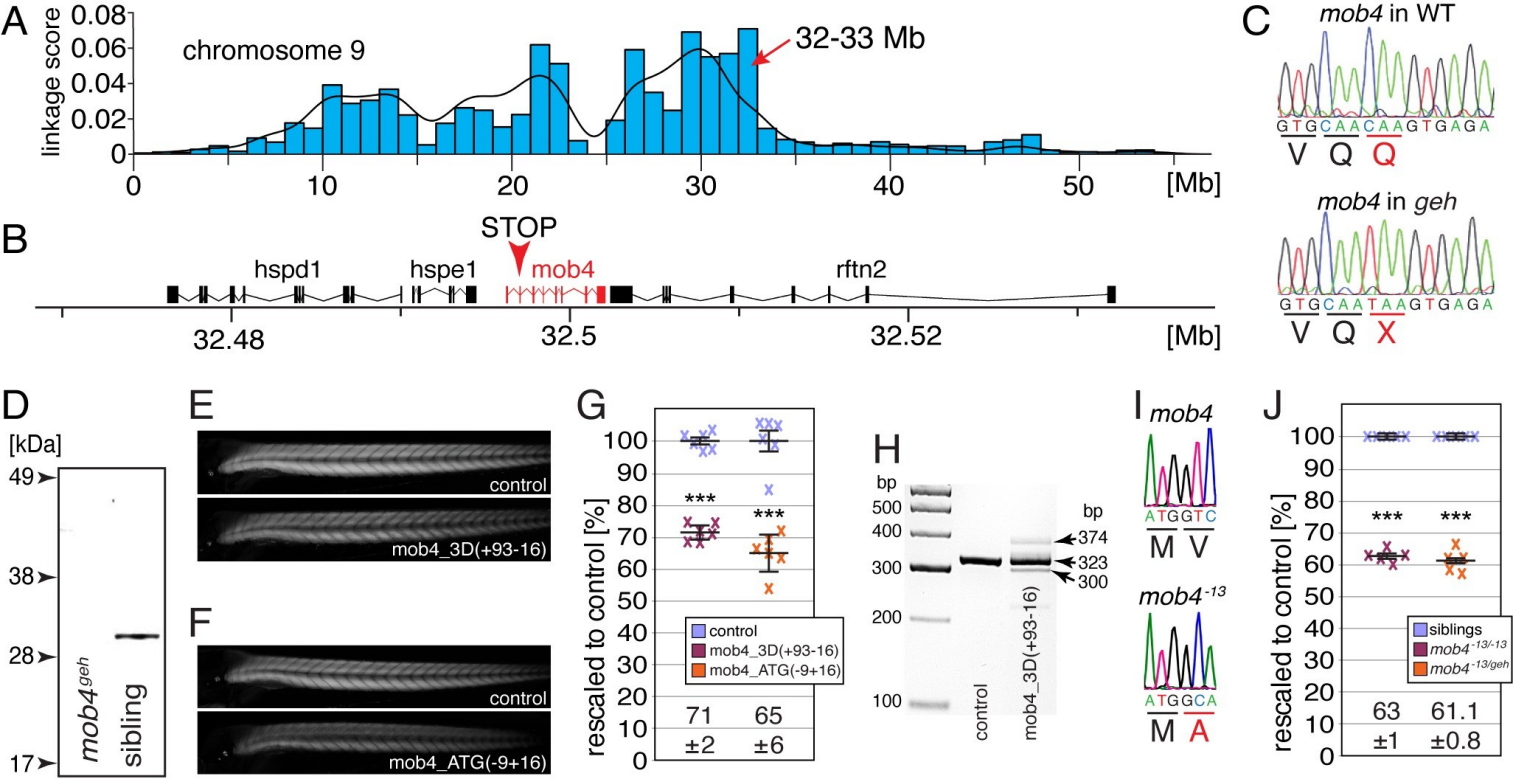
The function of *mob4* is lost within *gemütlich* mutants. (A) Linkage analysis of *gemütlich* revealed a region of homozygosity on chromosome 9 with a peak between 32 to 33 Mb. (B) The MOB family member 4 (*mob4*) gene was located within the linked region. (C) Genomic sequences show that the mutant *gemütlich* harboured a *mob4* allele with a premature stop codon in exon 2 (Q41X). (D) Western blot analysis using antibodies against human MOB4 showed epitope loss in *mob4*^*geh*^ homozygotes. (E) Knockdown of *mob4* by the morpholinos mob4_3D(+93–16) that targets the splice donor of exon 3 or (F) mob4_ATG(-9+16) that targets the translation start codon led to a reduction in birefringence. (G) Compared to control injected 3-dpf-old larvae (100 ± 1% and 100 ± 2%, respectively), administration of mob4_3D(+93–16) induced a reduction in birefringence to 71 ± 2% and mob4_ATG(-9+16) to 65 ± 6%. Crosses represent individual larvae (n = 6). (H) RT-PCR using primers targeting exons 1 and 5 of *mob4* revealed altered splicing in mob4_3D(+93–16)-injected larvae. (I) The *mob4*^*-13*^ allele harboured a genomic deletion of 13 bp from exon 1 (g.5_17del). (J) Compared to 3-dpf-old siblings (both 100 ± 1%), the birefringence of *mob4*^*-13*^ homozygotes and *mob4*^*-13/geh*^ compound heterozygotes was significantly reduced to 63 ± 1% and 61.1 ± 0.8%, respectively. Crosses represent averaged birefringence of clutches with a minimum of 6 larvae per genotype (n = 5 clutches). Data are presented as mean ± SEM; *** P < 0.001 calculated by Student’s t-test.

To identify the phenotype-causing mutation, *geh* mutants were subjected to positional cloning based on SNP analysis. The offspring a single *geh* mapping cross was sorted using birefringence analysis and the genomic DNA of pooled homozygotes and siblings was sequenced by next generation sequencing. Sequence variants were identified via the MiModD software. Regions of homozygosity were only found on chromosome 9 with the highest peak located between 32 to 33 Mb (Fig 2A). Subsequent sequence analysis discovered a single nucleotide change (from C to T) resulting in a nonsense mutation (Q41X) in exon 2 of *MOB family member 4* (*mob4*) located within the homozygosity region (Fig 2B and 2C). Sequence alignment of Mob4 from different species showed the protein’s high conservation and the position of the affected amino acid (S1 Fig). Other mutations, predicted to alter gene functions, were not found within the linked locus. Loss of Mob4 protein in *mob4*^*geh*^ homozygotes was confirmed by Western blot using antibodies against human MOB4 (Fig 2D). To confirm that the muscle phenotype of *mob4*^*geh*^ is induced by a mutated *mob4* allele, knockdown of *mob4* was performed with two independent morpholinos: the splice-altering morpholino mob4_3D(+93–16), which targets the splice donor of exon 3, and the translation-blocking morpholino mob4_ATG(-9+16). Administration of both morpholinos induced a significant birefringence reduction compared to control injected wildtypes, resembling the birefringence reduction of *mob4*^*geh*^ (Fig 2E, 2F and 2G). Functionality of mob4_3D(+93–16) was confirmed by RT-PCR that demonstrated the altered splicing of *mob4* within the morphants (Fig 2H). Furthermore, a second *mob4* mutant allele was generated by CRISPR/Cas9 technology. Administration of a single guidance RNA targeting exon 1 led to isolation of the *mob4*^*-13*^ mutant line that harboured a genomic deletion of 13 base pairs (bp) annotated as coding sequence located directly downstream of the ATG translation start of *mob4* (NM_001003439c.5_17del) (Figs 2I and S2). In comparison to their siblings, the birefringence of *mob4*^*-13*^ homozygotes as well as *mob4*^*13/geh*^ compound heterozygotes was significantly reduced, confirming that the muscle phenotype of *mob4*^*geh*^ can be attributed to the mutations within *mob4* (Fig 2J). Similar to *mob4*^*geh*^, *mob4*^*-13*^ homozygotes did not survive past 6 pdf.

In summary, the reduced birefringence of *mob4*^*geh*^ mutants is caused by loss of *mob4* function.

### Mob4 locates at the sarcomere’s Z-disc within cranial and trunk myofibres, where it is involved in the regulation of myofibril growth

**Fig 3:**
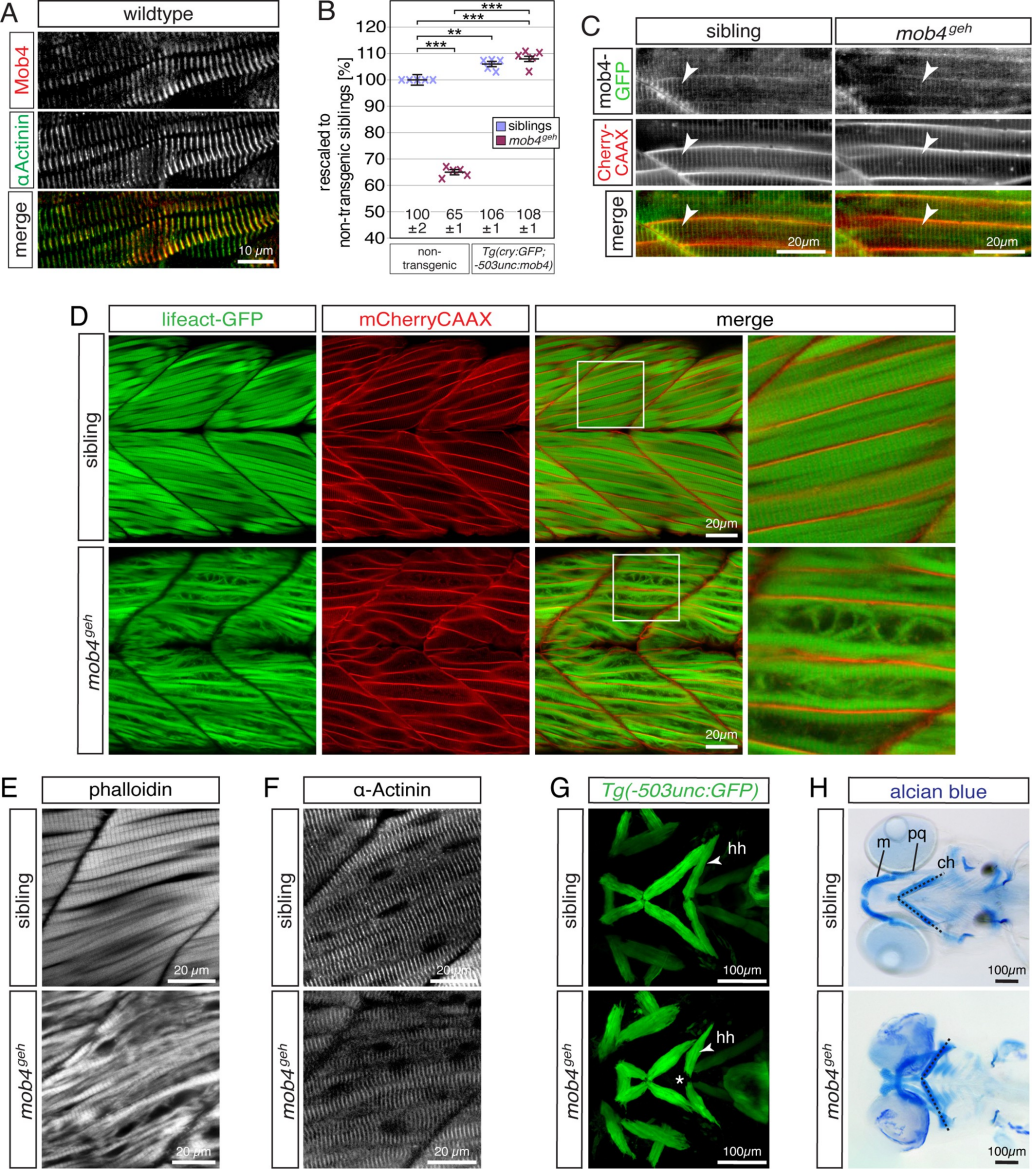
Mob4 is located at the Z-disk, where it might be involved in the regulation of myofibril assembly. (A) At 3dpf, antibodies against human MOB4 colocalised with antibodies against the Z-disc protein α-Actinin (n = 6 per genotype). (B) At 3 dpf, the birefringence of *mob4*^*geh*^ homozygotes was significantly higher in the transgenic background of *Tg(cry*:*GFP;-503unc*:*mob4)*. Also compared to non-transgenic siblings (100 ± 2%), the birefringence of *Tg(cry*:*GFP;-503unc*:*mob4)* transgenic *mob4*^*geh*^ homozygotes (108 ± 1%) and siblings (106 ± 1%) was significantly higher. Crosses represent averaged birefringence of clutches with a minimum of 4 larvae per genotype (n = 5 clutches). Data are presented as mean ± SEM; *** P < 0.001 and ** P < 0.01 by one-way ANOVA with post hoc Tukey’s test. (C) In 3-dpf-old siblings and *mob4*^*geh*^ homozygotes, Mob4-GFP fusion protein (green) expressed via *Tg(cry*:*GFP;-503unc*:*mob4-GFP)* colocalised to t-tubules (red, arrowhead) marked in the *Tg(acta1*:*mCherryCAAX)* transgenic background (n = 3 per genotype). (D) Highlighting F-actin with transgenic *Tg(acta1*:*lifeact-GFP)* in green confirmed residual myofibril striation and revealed disorganised thin filaments within *mob4*^*geh*^ homozygotes at 3 dpf. Sarcolemma and t- tubules were labelled by mCherry fluorescence (red) in the *Tg(acta1*:*mCherryCAAX)* transgenic background (n = 6 per genotype). Boxed areas are magnified. (E) Labelling of F-actin with phalloidin revealed that the robust myofibril striation of siblings was reduced in *mob4*^*geh*^ homozygotes at 3 dpf (n = 6 per genotype). (F) At 3 dpf, antibodies against α-Actinin that mark sarcomere’s Z-disks showed the typical striation of the myofibril in siblings and *mob4*^*geh*^ homozygotes (n = 4 per genotype). (G) Visualisation of the cephalic muscles in the transgenic *Tg(−503unc*:*GFP)* background revealed that, in contrast to siblings, a gap was formed between the two hyohyoideus (hh) muscles in *mob4*^*geh*^ homozygotes at 3 dpf (representative Z-stacks) (n = 3 per genotype). (H) At 6 dpf, representative Z-stack projections of Alcian blue stained larvae depicted cartilage malformations in *mob4*^*geh*^ homozygotes and a widened angle formed by the two ceratohyal cartilage structures (dotted lines) (n = 4 per genotype). Designations: ceratohyal (ch); Meckel’s cartilage (m); palatoquadrate (pq). Scale bar sizes are indicated.

To analyse whether Mob4 locates subcellularly at the myofibril, antibodies against human MOB4 were used on 3-dpf-old sagittal muscle sections. Within wildtype zebrafish, Mob4 protein localised to the sarcomere’s Z-discs, which were identified by co-localisation with antibodies against the Z-disc marker α-Actinin (Fig 3A). As expected from the Western blot (Fig 2D), MOB4 antibodies did not locate to a specific region in skeletal muscle of 3-dpf-old *mob4*^*geh*^ homozygotes (S3 Fig). To further confirm that *mob4* function is required for myofibril assembly, the transgenic line *Tg(cry*:*GFP;-503unc*:*mob4)* was generated that expressed transgenic *mob4* under the control of the muscle-specific *503unc* promoter [30]. Birefringence analysis at 3 dpf demonstrated that directed expression of *mob4* significantly rescued the birefringence of non-transgenic *mob4*^*geh*^ homozygotes, further verifying that the birefringence reduction of *mob4*^*geh*^ is caused by the mutant *mob4*^*geh*^ allele (Fig 3B). Interestingly, the birefringence of *Tg(cry*:*GFP;-503unc*:*mob4)* transgenic *mob4*^*geh*^ homozygotes and siblings was significantly higher compared to non-transgenic siblings, suggesting that an increase of Mob4 levels could result in an increase in the amount of organised myofibril. This important finding suggests that *mob4* function might be involved in the regulation of the amount of organised myofibril. To additionally verify the Z-disc location of Mob4 protein in live zebrafish, the transgenic line *Tg(cry*:*GFP;-503unc*:*mob4-GFP)* was generated to express the Mob4-GFP fusion protein in skeletal muscle. In-line with the immunohistochemical results, Mob4-GFP co-localised with t-tubules that coincide with the Z-disc and were marked with transgenic mCherry-CAAX in the transgenic background of *Tg(acta1*:*mCherryCAAX)* (Fig 3C). Importantly, functionality of GFP-tagged Mob4 was confirmed by the rescue of the birefringence reduction of *mob4*^*geh*^ mutants as revealed by birefringence analysis at 3 dpf (S4 Fig). Similar to the transgenic Mob4, forced activation of Mob4-GFP also resulted in a birefringence that was significantly higher compared to non-transgenic *mob4*^*geh*^ homozygotes and siblings.

To assess the sarcomere organisation within live *mob4*^*geh*^ mutants, *mob4*^*geh*^ was crossed into the transgenic background of *Tg(acta1*:*lifeact-GFP)* and *Tg(acta1*:*mCherryCAAX)*. The fusion protein Lifeact-GFP directed GFP fluorescence to thin filaments, and the sarcolemma as well as t-tubules were marked by the integration of mCherry-CAAX [31]. In-line with the previously documented reduction in birefringence, Lifeact-GFP detected residual myofibril striation within live *mob4*^*geh*^ homozygotes, but also highlighted isolated and misoriented thin filaments (Fig 3D). Quantification of the combined diameter of striated myofibril within myofibres confirmed that striated myofibril is significantly reduced within *mob4*^*geh*^ mutants (13.8 ± 0.3 μm in siblings and 8.7 ± 0.2 μm in homozygotes, n = 4, P < 0.01 calculated by Student’s t-test). To confirm the residual striation in *mob4*^*geh*^ homozygotes, the F-actin marker phalloidin was used to expose regular striated sarcomeres within *mob4*^*geh*^ mutants. As indicated by birefringence analysis, striation was severely reduced within *mob4*^*geh*^ mutants and, in contrast to the siblings, abundant isolated filaments were detected (Fig 3E). Further immunohistochemistry with antibodies against the Z-disc marker α-Actinin revealed that regular sarcomeric Z-discs are formed within *mob4*^*geh*^ mutants (Fig 3F).

To assess the cranial musculature of *mob4*-deficient zebrafish, *mob4*^*geh*^ homozygotes were crossed into the *Tg(-503unc*:*GFP)* transgenic background that marks myofibres with GFP fluorescence. Although the cranial musculature of *mob4*^*geh*^ homozygotes appeared anatomically comparable to siblings at 3 dpf, a gap was formed between the two contralateral hyohyoideus muscles within *mob4*^*geh*^ homozygotes (Fig 3G). Similarly, severe cartilage malformations were apparent in Alcian Blue stained *mob4*^*geh*^ homozygotes at 6 dpf, which is indicative of muscle weakness as muscle force is known to affect cartilage morphology (Fig 3H).

Taken together, myofibril assembly within the trunk muscle is compromised and the cranial musculature could be weakened within *mob4*^*geh*^ mutants. The abundance of isolated thin filaments further suggests a defective processing of thin filaments, which is in-line with the Mob4 localisation at Z-discs, where thin filament assembly is initiated. In addition, the enhanced birefringence after directed activation of *mob4* indicates that *mob4* function might be involved in the regulation of myofibril growth.

### Thin filament biogenesis in *mob4*^*geh*^ results in nemaline-like bodies

**Fig 4:**
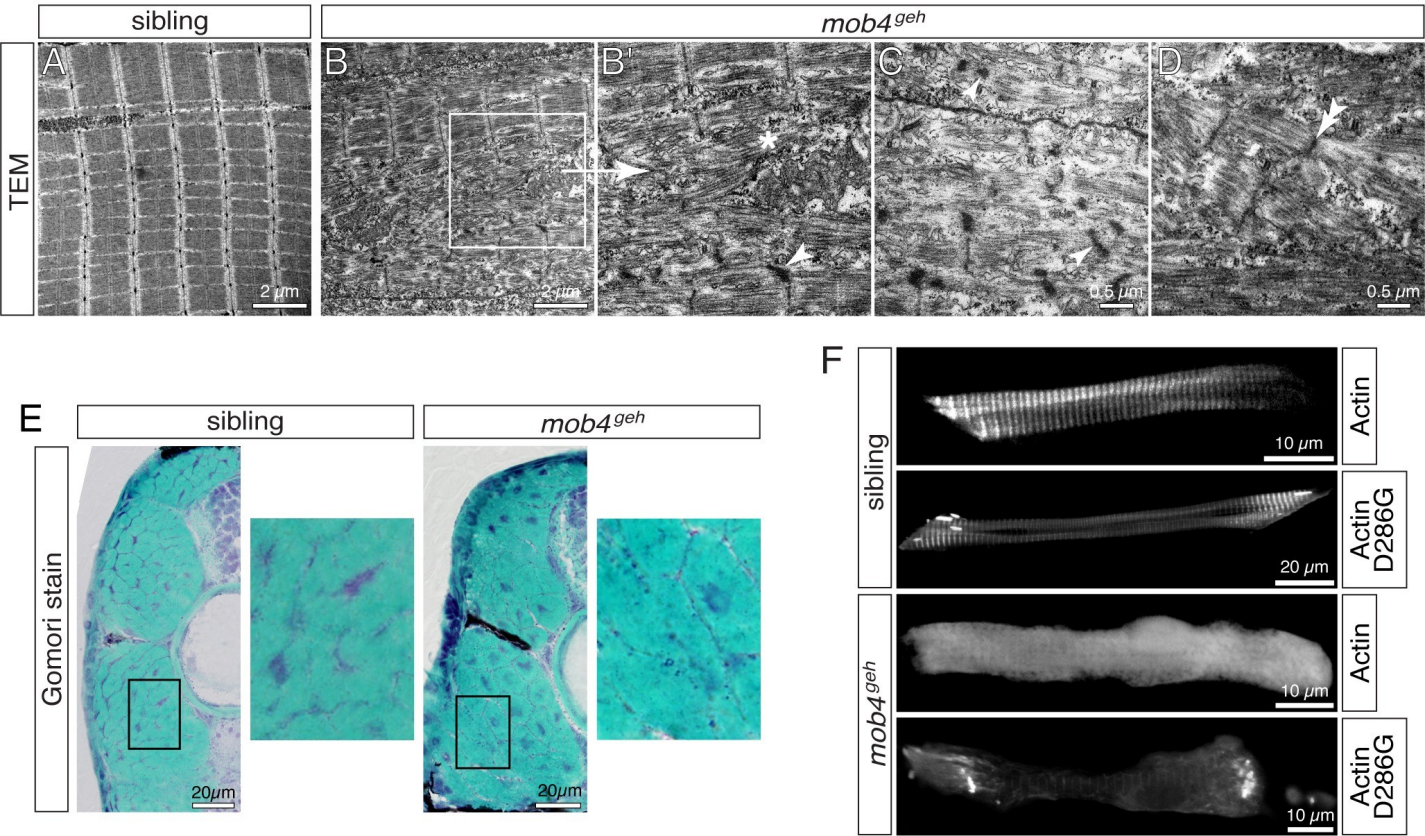
Sarcomere organisation is compromised in *mob4*^*geh*^ mutants. (A) Transmission electron micrograph depicted highly organised and arrayed myofibrils in 3-dpf-old siblings (n = 3). (B) Organised sarcomeres were rarely detected within *mob4*^*geh*^ homozygotes (n = 3). (B’) As shown in the magnification of the boxed area, sarcomeres were frequently disorganised and deposits of isolated filaments (asterisk) in addition to electron-dense structures (arrowhead), often associated with Z-disks, were found instead. (C) Electron-dense aggregates of *mob4*^*geh*^ homozygotes often showed a lattice structure (arrowhead) and (D) fragmented sarcomeres and widened Z-disks (double-arrowhead) were detected as well. (E) At 3 dpf, Gomori trichrome staining revealed subsarcolemmal dark blue structures within *mob4*^*geh*^ homozygotes but not siblings (n = 6 per genotype). Boxed areas are shown in higher magnification. (F) GFP fluorescence of transgenic ACTA1-GFP showed a striated pattern in 3-dpf-old siblings and a uniform pattern in *mob4*^*geh*^ homozygotes. Expression of ACTA1^D286G^-GFP led to rod-shaped structures in siblings and exclusively amorphic aggregates within *mob4*^*geh*^ homozygotes (n = 6 per genotype). Scale bar sizes are indicated.

To study the sarcomere organisation within *mob4*-deficient mutants in more detail, transmission electron microscopy (TEM) was performed. Consistent with previous results, residual organised sarcomeres, comparable to the ones within siblings, were found within 3-dpf-old *mob4*^*geh*^ homozygotes (Fig 4A and 4B). However, additional disorganised and fragmented sarcomeric structures along with isolated filaments were detected in *mob4*^*geh*^ mutants using TEM, indicating that sarcomere assembly is disrupted (Fig 4B, 4C and 4D). Furthermore, widened Z-discs and small electron-dense structures close to Z-discs of *mob4*^*geh*^ mutants were revealed by TEM (Fig 4B, 4C and 4D). Interestingly, the electron-dense aggregates of *mob4*^*geh*^ mutants often featured a lattice structure, which is characteristic for nemaline bodies that define human nemaline myopathy [32]. To further characterise the detected electron-dense aggregates, Gomori trichrome staining, a clinical marker for nemaline bodies [2], was performed on cross sections of 3-dpf-old larvae. In contrast to the siblings, subsarcolemmal structures were stained in dark blue in *mob4*^*geh*^ homozygotes (Fig 4E). Thus, although aggregates were not detected using α-Actinin antibodies and thin filament markers that typically mark nemaline bodies in humans, TEM and Gomori trichrome staining indicated the presence of nemaline-like bodies within *mob4*^*geh*^ mutants.

To further assess actin biogenesis and aggregate formation within *mob4*^*geh*^ mutants, fusion proteins of GFP with human skeletal α-actin (ACTA1-GFP) and a mutant isoform of α-actin (ACTA1^*D286G*^-GFP) were transiently expressed under the muscle-specific *503unc* promoter [30]. ACTA1-GFP has been shown to incorporate into sarcomeres of mice and zebrafish, whereas ACTA1^*D286G*^-GFP, which has been associated with nemaline myopathy in humans, has been reported to form rod-shaped nemaline bodies in addition to its sarcomere integration [33,34]. In 3-dpf-old siblings, the obtained striated GFP fluorescence pattern reported the expected ACTA1-GFP incorporation into sarcomeres (Fig 4F). In *mob4*^*geh*^ mutants, however, ACTA1-GFP was not incorporated into residual sarcomeres as shown by the uniform GFP fluorescence, indicating that α-actin processing is affected within *mob4*^*geh*^ mutants (Fig 4F). Expression of the mutant isoform ACTA1^D286G^-GFP in siblings led to the expected GFP fluorescence from GFP-positive striated sarcomeres and rod-shaped nemaline bodies. In *mob4*^*geh*^ homozygotes, however, GFP fluorescence visualised exclusively amorphic aggregates along with a faint striated pattern, demonstrating that *mob4* is also involved in the formation of rod-shaped nemaline bodies (Fig 4F).

In conclusion, *mob4* function is required for the incorporation of skeletal muscle α-actin into organised sarcomeres and loss of *mob4* function results in aggregates, which share only some aspects of nemaline bodies present in human nemaline myopathy.

### Mob4 interacts with TRiC to regulate myofibril growth

**Fig 5:**
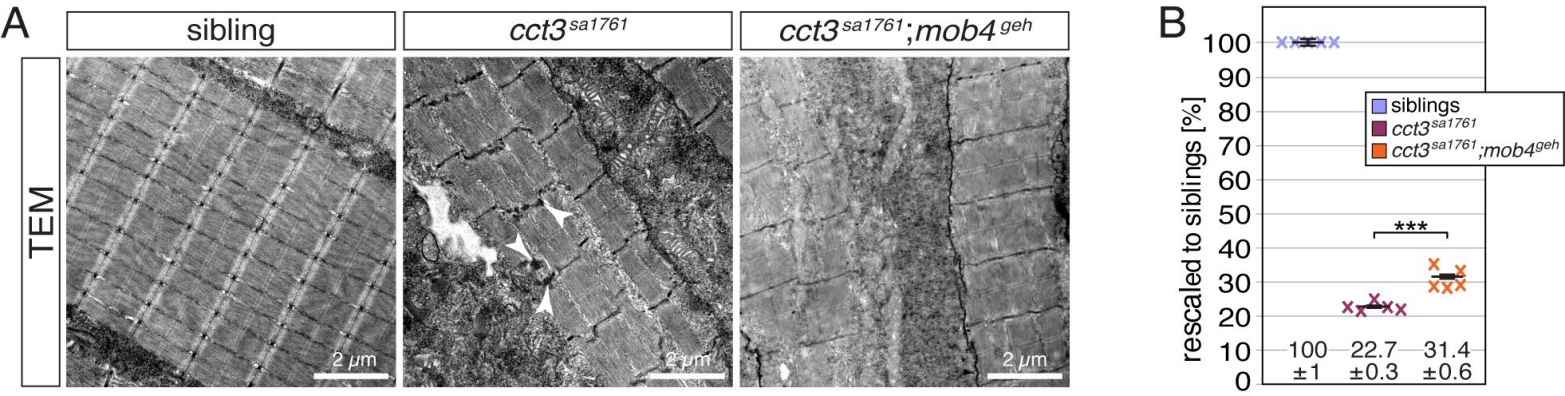
Mob4 interacts with TRiC. (A) As depicted in transmission electron micrographs, the sarcomere organisation detected in siblings was compromised in single *cct3*^*sa1761*^ homozygotes as well as *cct3*^*sa1761*^;*mob4*^*geh*^ compound homozygotes. Electron-dense aggregates (arrowhead) as found in *cct3*^*sa1761*^ homozygotes were absent in *cct3*^*sa1761*^;*mob4*^*geh*^ compound homozygotes (n = 3 per genotype). Scale bar sizes are 2 μm. (B) After rescaling to siblings (100 ± 1%), the birefringence of *cct3*^*sa1761*^;*mob4*^*geh*^ compound homozygotes (31.4 ± 0.6%) was significantly ameliorated compared to single *cct3*^*sa1761*^ homozygotes (22.7 ± 0.3%). Crosses represent averaged birefringence of clutches with a minimum of 4 larvae per genotype (n = 5 clutches). Data are presented as mean ± SEM; *** P < 0.001 by one-way ANOVA with post hoc Tukey’s test.

TRiC locates at the Z-disc similar to Mob4 and both single loss-of-function mutants, *mob4*^*geh*^ and *cct3*^*sa1761*^, are characterised by compromised α-actin biogenesis and nemaline-like body formation [8]. Furthermore, TRiC and Mob4 are part of a multiprotein complex, as shown by co-immunoprecipitation in human cells [35], in which they directly interact as demonstrated by genome-wide association studies in nematodes [36]. Co-localisation of Mob4 and TRiC was confirmed with antibodies against human MOB4 and human CCT5 (S5 Fig). In order to genetically evaluate the interaction of Mob4 with TRiC *in vivo*, *mob4*^*geh*^ was crossed to *cct3*^*sa1761*^, in which *cct3* deficiency results in loss of TRiC function [8]. At 3 dpf, TEM micrographs depicted highly organised sarcomeres in siblings and the expected nemaline-like aggregates in single *cct3*^*sa1761*^ homozygotes (Fig 5A), which were comparable in shape and location to the aggregates of *mob4*^*geh*^ (Fig 4B). However, compound *mob4*^*geh*^;*cct3*^*sa1761*^ homozygotes were devoid of electron-dense aggregates. To further evaluate this interesting finding, birefringence analysis was performed at 3 dpf. Analysis of single *cct3*^*sa1761*^ homozygous siblings resulted in the expected severe birefringence reduction [8], but the birefringence of *mob4*^*geh*^;*cct3*^*sa1761*^ homozygotes was significantly higher, demonstrating a significant amelioration of the *cct3*^*sa1761*^ birefringence reduction (Fig 5B). The increased birefringence of compound *mob4*^*geh*^;*cct3*^*sa1761*^ homozygotes, which were devoid of aggregates, over single mutants that featured aggregates, also suggests that aggregates could contribute to the severity of myopathies.

In summary, analysis of compound *mob4*^*geh*^;*cct3*^*sa1761*^ mutants, in combination with the previously established molecular interaction between MOB4 and TRiC in human cells [35] and our co-localization results, is consistent with the model that the two proteins interact *in vivo* in zebrafish as well, and that *mob4* function is required for the establishment of normal myofibril structure. Furthermore, compound *mob4*^*geh*^;*cct3*^*sa1761*^ mutants that were devoid of aggregates suggesting that aggregates could contribute to the severity of myopathies.

### Neuronal connectivity is compromised in *mob4*^*geh*^

**Fig 6:**
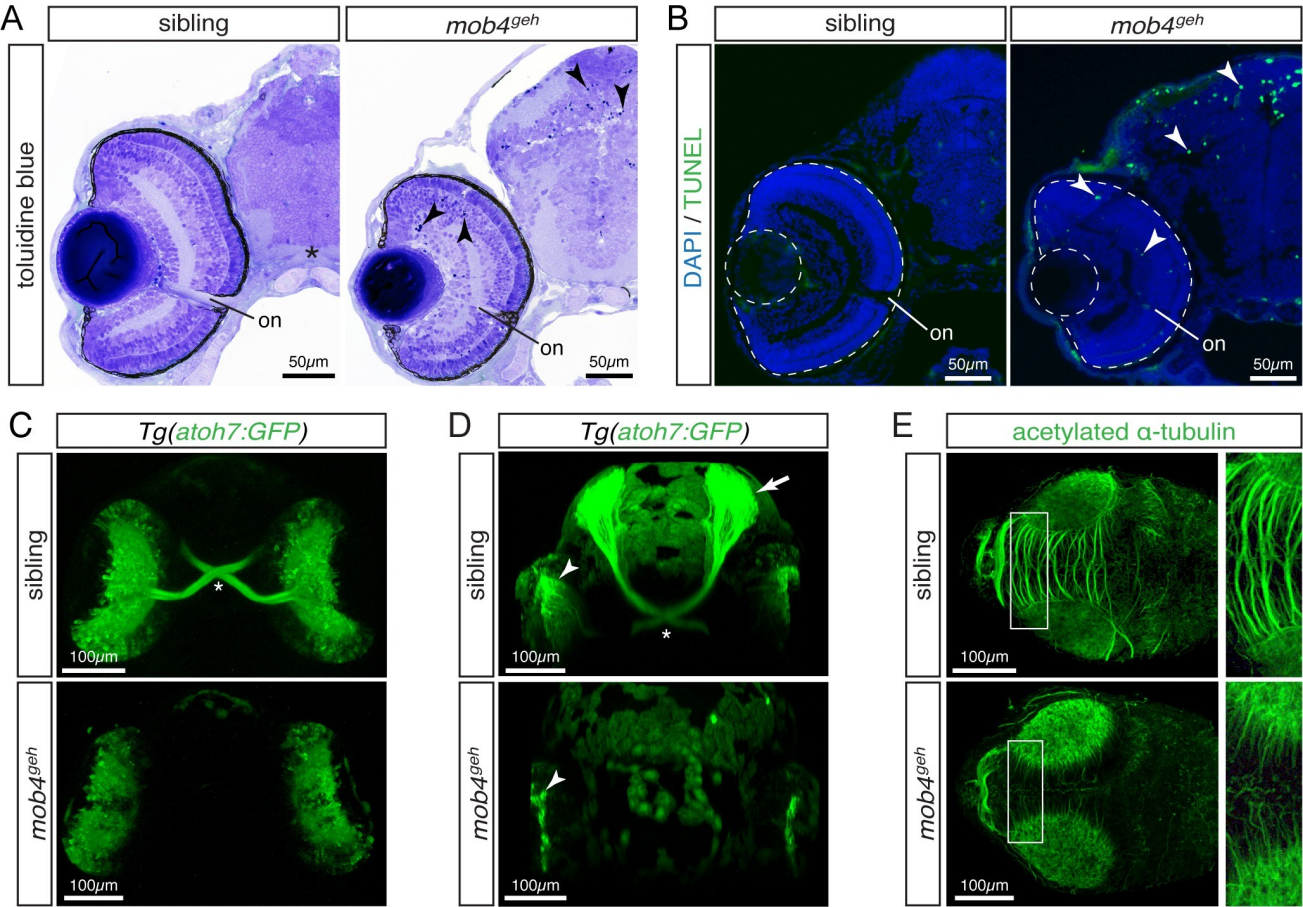
Loss of *mob4* function compromises neuronal connectivity. (A) At 3 dpf, toluidine blue-stained sections displayed pyknotic nuclei (arrowhead) dispersed throughout the retina and tectum of *mob4*^*geh*^ homozygotes, not siblings (n = 4 per genotype). (B) Abundant apoptosis within the retina and tectum of 3-dpf-old *mob4*^*geh*^ homozygotes was detected by TUNEL assay (n = 8 per genotype). (C) In representative ventral views (Z-stack), the optic chiasm (asterisk) was highlighted by *Tg(ath7*:*GFP)* within 3-dpf-old siblings but not *mob4*^*geh*^ homozygotes (n = 3 per genotype). (D) In representative Z-stacks, axons of retinal ganglion cell marked by *Tg(ath7*:*GFP)* project contralaterally via the optic chiasm (asterisk) from the retina (arrowhead) onto the tectum (arrow) of 3-dpf-old siblings (n = 4). In contrast, axons were not formed by retinal ganglion cells (arrowhead) of *mob4*^*geh*^ homozygotes (n = 4). (E) In Z-stacks of 3-dpf-old larvae, antibodies against acetylated α-tubulin revealed defective neurite formation within the tectum of *mob4*^*geh*^ homozygotes. Boxed areas are shown in higher magnification (n = 3 per genotype). Scale bar sizes are indicated.

*Drosophila* dMob4 has been reported to play a role in microtubule organization, which is essential for neurite branching and axonal transport [19]. Besides α-actin, TRiC also folds α- and β-tubulin and accordingly neuronal neurite formation is severely reduced in TRiC-deficient zebrafish [8,16]. To analyse neurons within *mob4*^*geh*^, cranial sections were stained with toluidine blue at 3 dpf. Although the size of the retina of *mob4*^*geh*^ homozygotes (23’800 ± 200 μm^2^) was significantly smaller compared to their siblings (25’000 ± 100 μm^2^) (S6 Fig), the morphology appeared largely comparable to their siblings (Fig 6A). However, in contrast to their siblings, pyknotic nuclei were detected dispersed throughout the retina and the tectum of *mob4*^*geh*^ homozygotes (Fig 6A). To study the cell death in more detail, Terminal deoxynucleotidyl transferase dUTP nick-end labelling (TUNEL) staining was performed on cryopreserved sections to detect apoptosis. Whereas apoptotic signals were barely detected in 3-dpf-old siblings, abundant apoptotic cells were present within the retina and tectum of *mob4*^*geh*^ homozygotes (Fig 6B). Neuron viability depends on neurites to innervate their target tissues and the axons of the retinal ganglion cells project through the optic chiasm to the contralateral tectum. To highlight retinal ganglion cells with GFP fluorescence in live zebrafish, *mob4*^*geh*^ was crossed into the transgenic background of *Tg(atoh7*:*GFP)* [37]. Consistent with the tectonal and retinal apoptosis, retinal ganglion cells of 3-dpf-old siblings were found to project via the optic chiasm to the optic tectum, however within *mob4*^*geh*^ homozygotes only somas of retinal ganglion cells were detected (Fig 6C and 6D). To assess the microtubule network required for axon formation, immunostaining with antibodies against acetylated α-tubulin was performed. In contrast to the siblings, a severe reduction in tectonal neurites was detected within *mob4*^*geh*^ homozygotes and the intertectal commissure was completely absent, indicating that microtubules are compromised within *mob4*^*geh*^ (Fig 6E). The intertectal commissure was also lost in *mob4*^*-13*^ homozygotes (S7 Fig), suggesting that in addition to the muscle phenotype also the neuronal phenotype of *mob4*^*-13*^ homozygotes phenocopied *mob4*^*geh*^ mutants.

In conclusion, it could be speculated that the compromised microtubule network within *mob4*-deficient zebrafish could lead to the severely reduced axon formation of retinal ganglion cells, which could consequently result in the detected retinal and tectonal apoptosis and the diminished retina size.

### Loss of *strn3* function phenocopies *mob4* deficiency

**Fig 7:**
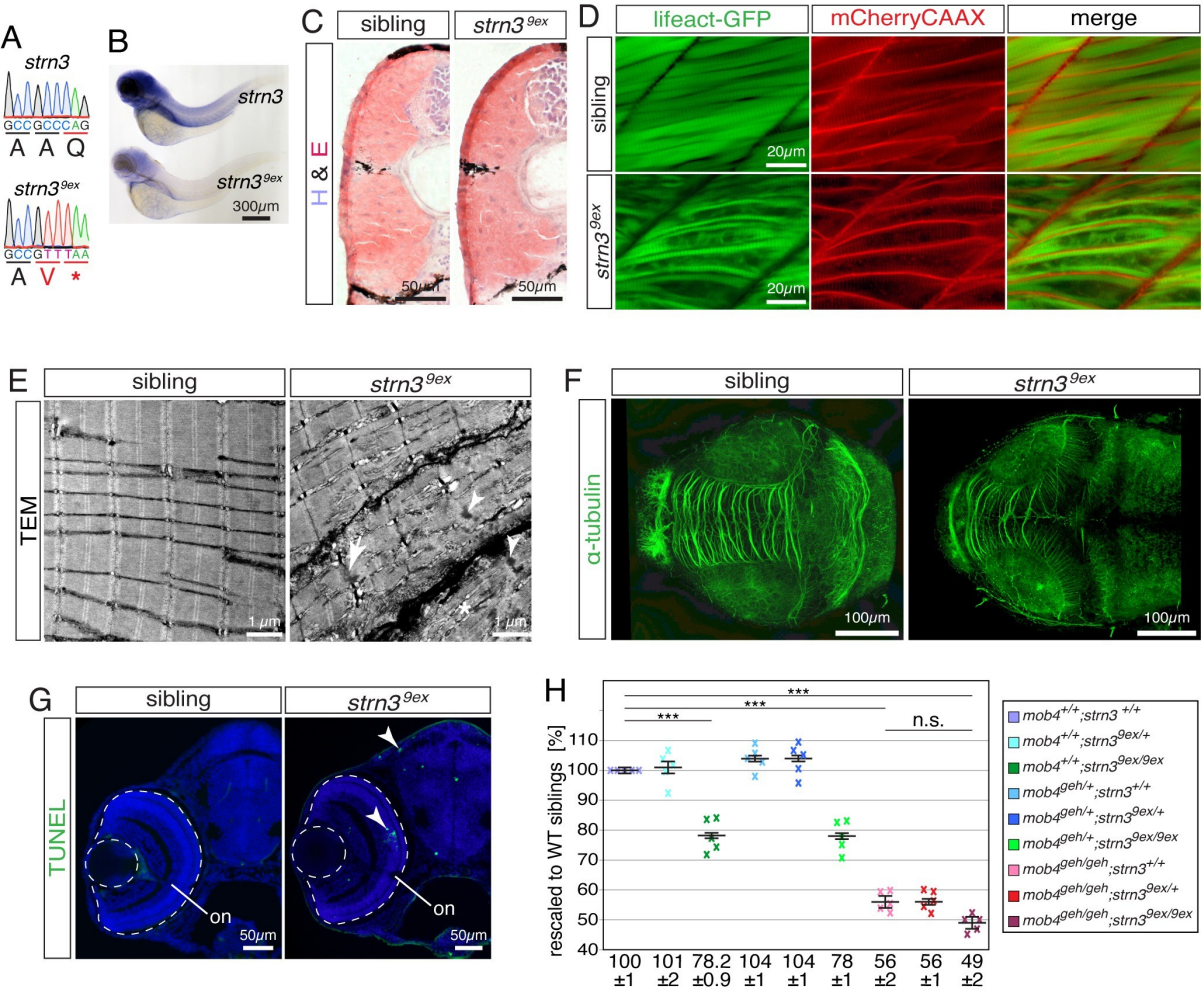
Loss of *strn3* phenocopies *mob4*-deficient zebrafish. (A) The genomic sequence of the *strn3*^*9ex*^ allele harboured a deletion of 23,516 bp and simultaneous insertion of 35 bp. (B) In comparison to 3-dpf-old siblings, the signal intensity of the *in situ* hybridisation using a *strn3* probe was strikingly diminished in *strn3*^*9ex*^ homozygotes (n = 5 per genotype). (C) Fibrotic signs were not detected on representative cross sections of 3-dpf-old *strn3*^*9ex*^ homozygotes and siblings stained with H&E (n = 6 per genotype). (D) The transgenic marker lines *Tg(acta1*:*lifeact-GFP)* and *Tg(acta1*:*mCherryCAAX)* revealed that the sarcomere striation of live 3-dpf-old siblings was strikingly reduced in *strn3*^*9ex*^ homozygotes, which featured disorganised thin filaments instead (n = 7 per genotype). (E) The sarcomere organisation shown in representative transmission electron micrographs of 3-dpf-old siblings was frequently compromised in *strn3*^*9ex*^ homozygotes. Instead, *strn3*^*9ex*^ homozygotes featured widened Z-disks (arrow), filament deposits (asterisk) and lattice-patterned electron-dense structures (arrowheads) (n = 3 per genotype). (F) Representative Z-stacks of 3-dpf-old larvae stained with antibodies against acetylated α-tubulin. In contrast to their siblings, the intertectal commissure of *strn3*^*9ex*^ homozygotes was strikingly reduced with less neurites projecting across tectal lobes (n = 5 per genotype). (G) Enhanced apoptosis within the retina and tectum of 3-dpf-old representative *strn3*^*9ex*^ homozygotes was confirmed by TUNEL assay (n = 12 per genotype). (H) At 3 dpf, the birefringence of single *strn3*^*9ex*^ homozygotes (78.2 ± 0.9%) and *mob4*^*geh*^ homozygotes (56 ± 2%) as well as *strn3*^*9ex*^;*mob4*^*geh*^ compound homozygotes (49 ± 2%) was significantly reduced compared to their WT siblings (100 ± 1%). Importantly however, the birefringence of *strn3*^*9ex*^;*mob4*^*geh*^ compound homozygotes was not significantly reduced compared to their single *mob4*^*geh*^ homozygous siblings. Crosses represent averaged birefringence of clutches with a minimum of 4 larvae per genotype (n = 5 clutches). Data are presented as mean ± SEM; *** P < 0.001 calculated by Student’s t-test. Scale bar sizes are indicated.

In humans, MOB4 is a core component of the STRIPAK complex that uses striatin-3 (STRN3) as a scaffold [38]. To assess whether STRIPAK deficiency results in a phenotype comparable to *mob4*-deficient zebrafish, *strn3* was mutated using CRISPR/Cas9 technology. Simultaneous targeting of exons 1 and 9 of *strn3* with single guidance RNAs resulted in isolation of the *strn3*^*9ex*^ allele that harboured an insertion of 35 bp and simultaneous deletion of 23,516 bp at the genomic *strn3* locus (Figs 7A and S8). As a consequence, 1,234 bp were removed from the 2,157 bp annotated *strn3* coding sequence and 35 bp were added (NM_001013266c.65_1298delins35). The *strn3* gene locus expresses a single transcript according to the current genome assembly GRCz11, and thus the frame shifting *strn3*^*9ex*^ mutation is predicted to induce multiple pre-mature stop codons. To assess if the *strn3*^*9ex*^ transcript was subjected to nonsense-mediated decay, *in situ* hybridisation was performed with a *strn3* probe. At 3 dpf, the *in situ* hybridisation signal of *strn3*^*9ex*^ homozygotes was strikingly reduced compared to their siblings, revealing that *strn3*^*9ex*^ transcripts were degraded and indicating that the *strn3* function is lost in *strn3*^*9ex*^ mutants due to nonsense-mediated decay (Fig 7B). In contrast to their viable siblings, *strn3*^*9ex*^ homozygotes did not survive past 11 dpf. Starved siblings also died at 11 dpf, suggesting that a weakened musculature could leave *strn3*^*9ex*^ mutants unable to hunt for food effectively. Similar to *mob4*^*geh*^ mutants, fibrotic signs were not noticed on H&E-stained sections of 3-dpf-old *strn3*^*9ex*^ homozygotes (Fig 7C) and their CSA were comparable to siblings (0.0269 ± 0.0006 mm^2^ for *strn3*^*9ex*^ and 0.0276 ± 0.0003 mm^2^ for siblings; n = 5, P = 0.36 calculated by Student’s t-test).

Striated myofibril together with abundant disorganised thin filaments were also apparent within live *strn3*^*9ex*^ homozygotes in the transgenic background of *Tg(acta1*:*lifeact-GFP)* and *Tg(acta1*:*mCherryCAAX)* (Fig 7D). The combined diameter of striated myofibril within myofibres was significantly reduced within *strn3*^*9ex*^ mutants (9.9 ± 0.2 μm) in comparison to the siblings (13.9 ± 0.2 μm) (n = 4, P < 0.001 calculated by Student’s t-test). Accordingly, TEM revealed that 3-dpf-old *strn3*^*9ex*^ homozygotes featured disorganised sarcomeric structures with widened Z-discs and lattice-pattered electron-dense aggregates alongside organised sarcomeres (Fig 7E). Similar to *mob4*^*geh*^, the electron-dense aggregates detected by TEM were not marked by transgenic lifeact-GFP. Thus, *strn3*^*9ex*^ matched the muscle phenotype of *mob4*^*geh*^ mutants. To study the neuronal phenotype caused by *strn3* deficiency, immunohistochemistry with antibodies against acetylated α-tubulin was performed, which demonstrated that the intertectal commissure is severely reduced in *strn3*^*9ex*^ homozygotes compared to their siblings (Fig 7F). Accordingly, abundant apoptotic cells were detected by TUNEL assay in the retina and the tectum 3-dpf-old *strn3*^*9ex*^ homozygotes in contrast to their siblings (Fig 7G). Therefore, also the neuronal defects of *strn3*^*9ex*^ homozygotes matched the phenotype of *mob4*^*geh*^, although differences in severity levels were noted as the intertectal commissure is completely lost in *mob4*^*geh*^ and only reduced in *strn3*^*9ex*^. To genetically assess the interaction of Mob4 and Strn3, which has been shown in human [38], *strn3*^*9ex*^;*mob4*^*geh*^ compound mutants were generated and their muscle integrity analysed by birefringence assay. Similar to single *mob4*^*geh*^ mutants, the birefringence of single *strn3*^*9ex*^ homozygotes was significantly reduced compared to their WT siblings (Fig 7H), although the birefringence of *mob4*^*geh*^ was further reduced compared to *strn3*^*9ex*^ homozygotes. As expected, also the birefringence reduction quantified for *strn3*^*9ex*^;*mob4*^*geh*^ compound homozygotes was significant in comparison to WT siblings. However, in comparison to single *mob4*^*geh*^ homozygotes, the birefringence of compound homozygotes was not significantly changed. Thus, the birefringence analysis of *strn3*^*9ex*^;*mob4*^*geh*^ compound homozygotes is in accordance with previous results showing that human MOB4 and STRN3 are core components of the STRIPAK complex [38].

In summary, the muscular and the neuronal phenotype of both *strn3*^*9ex*^ and *mob4*^*geh*^ mutants share similar characteristics. Birefringence analysis of *strn3*^*9ex*^;*mob4*^*geh*^ compound mutants, in combination with the previously demonstrated *in vitro* evidence for interactions in human cells as core components of the STRIPAK complex [17,38], is consistent with the model that Mob4 and Strn3 interact in zebrafish muscle as well.

## Discussion

To discover novel molecules involved in vertebrate sarcomere assembly, a genetic screen was performed in zebrafish that resulted in the isolation of the mutant *gemütlich*, which featured sarcomere defects caused by loss of *mob4* function. Interestingly, electron-dense aggregates were found within *mob4*^*geh*^ that shared characteristics typical for nemaline bodies, namely their lattice structure, location close to actinin-rich Z-discs and Gomori trichrome stainability. However, in contrast to nemaline bodies that can be marked by antibodies against actinin and actin also in zebrafish [14,32], neither phalloidin nor antibodies against actinin were able to detect the *mob4*^*geh*^ aggregates. Further analysis utilising the nemaline-specific ACTA1^D286G^ isoform of α-actin [33], revealed that although *mob4*-deficiency induces formation of aggregates, *mob4* function might be required for the specific formation of nemaline bodies. These findings together with the notion that interaction of Mob4 with TRiC supresses the formation of amyloid β-rich plaques associated with Alzheimer’s disease [36], suggest that *mob4*^*geh*^ aggregates might represent a new class of aggregates. In addition, analysis of *cct3*^*sa1761*^;*mob4*^*geh*^ compound mutants demonstrated that removal of aggregates might ameliorate the muscle integrity, thus indicating that aggregates within skeletal muscle could be pathogenic. The direct interaction of Mob4 protein with TRiC demonstrated to occur in nematodes [36], appears to have a genetic correlate in zebrafish as suggested by the analysis of *cct3*- and *mob4*-deficient compound mutants showing that Mob4 genetically interacts with TRiC that folds α-actin during thin filament assembly [8]. Additionally, forced expression of *mob4* was not only able to restore the muscle integrity of *mob4*^*geh*^ mutants, but also significantly increased the birefringence, indicating that the amount of myofibril in wildtype zebrafish was increased. These results, together with our compound mutant analyses and previous reports [35,36], indicate that Mob4 and TRiC interact and suggest the possibility that Mob4 might regulate TRiC function to coordinate sarcomere assembly and growth.

The other main targets of TRiC apart from actin are α- and β-tubulin [16]. Comparable to the TRiC loss-of-function mutants, in which disruption of the microtubule networks leads to neuronal neurite degeneration and tectonal as well as retinal apoptosis [8], *mob4*^*geh*^ mutants were characterised by a defective microtubule network, impaired neurite formation and apoptosis. Moreover, previous studies in flies reported a role of Mob4 in axonal microtubule organization and focusing of microtubule-based spindles [19,20]. Thus, it could be speculated that, in addition to the sarcomere defects, also the neural defects of *mob4*^*geh*^ could be attributed to TRiC, such that Mob4 deficiency leads to TRiC dysregulation resulting in an impaired tubulin biogenesis and compromised microtubulin network and defective neurites.

Mob4 is a core component of the macromolecular STRIPAK complex that uses striatin proteins as scaffold [17,38]. Accordingly, *strn3*-deficient mutants featured neuronal as well as sarcomeric defects that reflected the characteristics of *mob4*-deficient mutants and *mob4*^*geh*^;*strn3*^*9ex*^ compound mutant analysis showed their interaction. Analyses of the birefringence, survival and intertectal commissures revealed that the *mob4*^*geh*^ phenotype was stronger compared to *strn3*^*9ex*^. Different striatins can be found within STRIPAK whereas Mob4 is a core protein of this complex [17]. Hence, the weaker phenotype of *strn3*^*9ex*^ could be attributed to Strn3 being replaced by other striatin proteins. Nonetheless, *strn3*^*9ex*^ mutant analysis together with the notion that Mob4 is a core component of STRIPAK suggests that Mob4 may function through its role within STRIPAK, also in zebrafish. Hence, the interaction of Mob4 with TRiC might indicate that Mob4 may be the link that connects the TRiC and STRIPAK complexes. However, confirmation of such a role will require further biochemical and *in vivo* cell biological studies to complement the genetic analyses we describe here.

## Material and methods

### Ethics statement

The Monash Animal Service approved the treatment of zebrafish males with N-ethyl-N-nitrosourea (MAS/2009/05) and the maintenance of zebrafish lines (ERM/22161).

### Data and software availability

All numeric data presented within this article have been placed into the supporting information S1 Data.

### Generation and genotyping of zebrafish lines and morphants

For the genetic screen, 48 adult male zebrafish in TU background were treated with N-ethyl-N-nitrosourea as described earlier [28]. Once mutagenized, F0 males were out-crossed over two generations and screening of 126 F2 families for a reduction in birefringence led to the identification of the *gemütlich* mutant, which was out-crossed over 9 generations to reduce background mutations before experiments were commenced. Genotyping of *mob4*^*geh*^ was achieved by using the oligonucleotides mob4_Hinc_F (5’-gaaatggacagcaccttggctggtcaa-3’) and mob4_1R (5’-ttcaaacaaaacagtgtaaacaacca-3’) in a PCR followed by restriction digestion with HincII. Generation of the CRISPR/Cas9 mutants was accomplished by micro-injection of Cas9, tracrRNA and crRNAs (IDT) into wildtype eggs using standard methods [8]. To generate *mob4*^*-13*^, a crRNA (5’- tgtcgagatggtcatggcggAGG) targeting exon 1 was used and *strn3*^*9ex*^ was generated by simultaneous injection of two crRNAs (5’-gaggaggaatggccgcccagAGG) and (5’-ttttggcaggaacggtcttgTGG) targeting exons 1 and 9, respectively. To subsequently identify genomic alterations within *mob4*, amplicons generated with the oligonucleotides mob4_2F (5’-cgcgctgtcaatcaaactct-3’) and mob4_2R (5’-ggatgtgctccacgaacagg-3’) were cloned into pCR2.1 (Life Technologies) and sequenced. Similarly, for *strn3* the oligonucleotides strn3_F (5’-gaggaggaggaggtggtggt-3’) and strn3_R (5’-tgggcatctcgtcttgctgt-3’) were utilised. To genotype *mob4*^*-13*^ and *strn3*^*9ex*^ by PCR, the oligonucleotides mob4_2F and mob4_2R or strn3_F and strn3_R were used, respectively. Morphants of *mob4* were generated with morpholino oligomers ordered from Gene Tools LLC (Philomath, USA). 1.4 nl of 40 μM mob4_ATG(-9+16) (5’-cctccgccatgaccatctcgacaga), 200 μM mob4_3D(+93–16) (5’-cagactaataatatacctgagatgt) and equimolar concentrations of standard control morpholino were micro-injected into wildtype eggs. Using the Gateway cloning system, the expression plasmids to generate *Tg(cry*:*GFP;-503unc*:*mob4)* and *Tg(cry*:*GFP;-503unc*:*mob4-GFP)* were combined using p5E-unc503, p3E-polyA and pDestTol2pACryGFP together with pME-mob4 and pME-mob4GFP, respectively [30]. Generation of the corresponding transgenic lines was approved by IBC/24002. Transient expression of ACTA1-GFP and ACTA1^D286G^-GFP was achieved by micro-injection of the plasmids pCryGFP;-503uncACTA1GFP and pCryGFP;-503uncACTA1^D286G^GFP [8]. All zebrafish lines were maintained in the TU (Tübingen) background.

### Linkage analysis

The mapping cross was established by crossing *geh* mutants in the TU background with wildtypes in the WIK background. The offspring of a single mapping pair was sorted using birefringence analysis and 50 siblings and 50 homozygotes were pooled separately. The genomic DNA of each pool was isolated and sequenced on an Illumina HiSeq 100-bp paired-end sequencer (Illumina, USA). Sequencing reads were aligned separately to the zebrafish Ensembl genome assembly GRCz10 with BAM and SAM tools. Subsequently, sequence variants were identified by the MiModD pipeline and used to generate a linkage map with the NacreousMap software integrated in MiModD [39]. Gene sequences within the homozygosity region were displayed and assessed for mutations using the Integrative Genome Viewer (IGV).

### Birefringence quantification

Individual zebrafish larvae were anaesthetised to prevent muscle contraction and automatically imaged with the microscope Abrio LS2.2 in an unbiased way. Subsequently, images were analysed as described earlier [27,40]. In short, the first 20 somites of imaged larvae were selected and the mean of all grey values of the pixels was measured utilising the software ImageJ. Obtained grey values were rescaled to siblings set to 100%. For analyses of single mutants, 5 clutches with a minimum of 6 larvae per genotype were assessed. Compound and transgenic mutants were analysed using 5 clutches with a minimum of 4 larvae per genotype and morphants were evaluated based on 6 individual larvae.

### Statistical analysis

Significance between two groups was determined by Student’s t-test and for multiple groups one-way ANOVA with post hoc Tukey’s test was used. Statistical significance was calculated by the software Prism (GraphPad Software). Presented data are mean ± standard error of the mean (SEM), calculated utilizing error propagation.

### Immunohistochemistry, histology, western blotting and RT-PCR

Western blotting, immunohistochemistry, TEM as well as the stains with H&E, Alcian Blue and Gomori trichrome were performed according to standard methods. Correct protein transfer during the Western blot analysis was confirmed by Ponceau S (Sigma) staining. Phalloidin was conjugated with AlexaFluor-568 (A12380, Life Technologies) and the primary antibodies were against α-Actinin (A7811, Sigma), MOB4 (HPA044125, Sigma), CCT5 (GTX110167, GeneTex) and acetylated α-tubulin (T6793, Sigma). Fluorescence images were recorded on a Zeiss LSM 510 Meta fluorescence confocal microscope (Zeiss, Germany) and electron micrographs on a Hitachi H7500 transmission electron microscope (Hitachi, Japan). RT-PCR with mob4_3D(+93–16) morphants was performed using mob4_3R (5’-atcaatggcagggcactctt) for reverse transcription of *mob4* transcript, followed by PCR with mob4_3F (5’-ggcacagctgttctgaggag) and mob4_4R (5’-tgggcagcacacagaaagat) targeting exons 1 and 5, respectively.

### Measurement of myofibril diameter

A confocal z-stack visualised striated myofibril within individual myofibres in the double-transgenic background of *Tg(acta1*:*mCherryCaaX)* and *Tg(acta1*:*liveact-GFP)*. Using the ImageJ software, the combined myofibril diameters in the middle of 10 individual myofibres were measured and averaged. 4 larvae per genotype were analysed (n = 4 per genotype) and statistical significance was determined via Student’s t-test.

## Supporting information

S1 Fig Alignment of Mob4 from different species. Alignment of the amino acid sequence of Mob4 from zebrafish (*Danio rerio*, D. r.), human (*Homo sapiens*, H. s.), mouse (*Mus musculus*, M. m.), fly (*Drosophila melanogaster*, D. m.) and nematode (*Caenorhabditis elegans*, C. e.) shows the high conservation of Mob4. Within *mob4*^*geh*^, the codon for glutamine at position 41 (red) was mutated into a pre-mature stop codon. In the *mob4*^*-13*^ allele 13 bp of coding sequence were deleted, constituting a frameshift mutation affecting the codons for the amino acids marked in grey and replacing the codon for glycine at position 6 (cyan) with a pre-mature stop codon. The peptide sequence of the immunogen of the antibodies against human MOB4 (HPA044125, Sigma) is underlined.
(TIF)

S2 Fig The *mob4*^*-13*^ allele harbours a 13 bp deletion. The larger context of the genomic DNA sequence of the mutation within *mob4*^*-13*^ shows that 13 bp (underlined) from the wildtype *mob4* are deleted in *mob4*^*-13*^ mutants.
(TIF)

S3 Fig Mob4 is not detected in *mob4*^*geh*^ mutants. At 3 dpf, antibodies against human MOB4 (HPA044125, Sigma) did not show a specific localisation within skeletal muscle of representative *mob4*^*geh*^ homozygotes (n = 4). Dotted lines indicate somite borders. Scale bar size is 20 μm.
(TIF)

S4 Fig Expression of the Mob4-GFP fusion protein rescued the birefringence reduction of *mob4*^*geh*^ mutants. At 3 dpf, birefringence analysis revealed that directed expression of the Mob4-GFP fusion protein in the muscle via *Tg(cry*:*GFP;-503unc*:*mob4-GFP)* significantly rescued the birefringence reduction of non-transgenic *mob4*^*geh*^ homozygotes. Furthermore, the birefringence of *mob4*^*geh*^ homozygotes (108 ± 2%) and siblings (105 ± 1%) within the *Tg(cry*:*GFP;-503unc*:*mob4-GFP)* transgenic background was significantly higher compared to non-transgenic siblings (100 ± 1%). Crosses represent averaged birefringence of clutches with a minimum of 4 larvae per genotype (n = 5 clutches). Data are presented as mean ± SEM; *** P < 0.001 and ** P < 0.01 by one-way ANOVA with post hoc Tukey’s test.
(TIF)

S5 Fig Mob4 co-localises with Cct5. Within representative 3-dpf-old wildtype larvae, antibodies against human MOB4 (HPA044125, Sigma) co-localised with antibodies against human CCT5 (GTX110167, GeneTex) (n = 6). Scale bar size is 20 μm.
(TIF)

S6 Fig The retina of *mob4*^*geh*^ mutants is reduced in size. Whereas the average size of the retina of siblings was 25’000 ± 100 μm^2^, the retina of *mob4*^*geh*^ homozygotes was significantly reduced to 23’800 ± 200 μm^2^. Crosses represent single retina sizes measured at the level of the optic nerve (n = 10). Data are presented as mean ± SEM; *** P < 0.001 by Student’s t-test.
(TIF)

S7 Fig The intertectal commissure is absent in *mob4*^*-13*^. Representative Z-stacks projections of 3-dpf-old larvae immunostained with antibodies against acetylated α-tubulin documented the absence of the intertectal commissure was within *mob4*^*-13*^ homozygotes (n = 6 per genotype). Scale bar sizes are 100 μm.
(TIF)

S8 Fig The *strn3*^*9ex*^ allele harbours an insertion of 35 bp and simultaneous deletion of 23,516 bp. The larger context of the genomic DNA sequence of the mutation within *strn3*^*9ex*^ revealed an insertion of 35 bp (underlined) and simultaneous deletion of 23,516 bp from the wildtype *strn3* allele.
(TIF)

S1 Video Touch-evoked response of 3-dpf-old *mob4*^*geh*^ homozygotes. In dishes of 9 cm diameter, the motility and forward thrust was impaired in 3-dpf-old *mob4*^*geh*^ homozygotes. Representative of 4 analysed homozygotes is shown (n = 4).
(MOV)

S2 Video Touch-evoked response of 3-dpf-old *mob4*^*geh*^ sibling. In dishes of 9 cm diameter, 3-dpf-old siblings exerted a robust motility and forward thrust. Representative of 4 analysed homozygotes is shown (n = 4).
(MOV)

S1 Data Raw data of values presented in graphs within figures. (XLSX)
