# Supplementary figures and images for: Mob4-dependent STRIPAK involves the chaperonin TRiC to coordinate myofibril and microtubule network growth
Source: PLoS Genet. 2022 Jun 23;18(6):e1010287. doi: 10.1371/journal.pgen.1010287 (PMC9258817; doi:10.1371/journal.pgen.1010287)

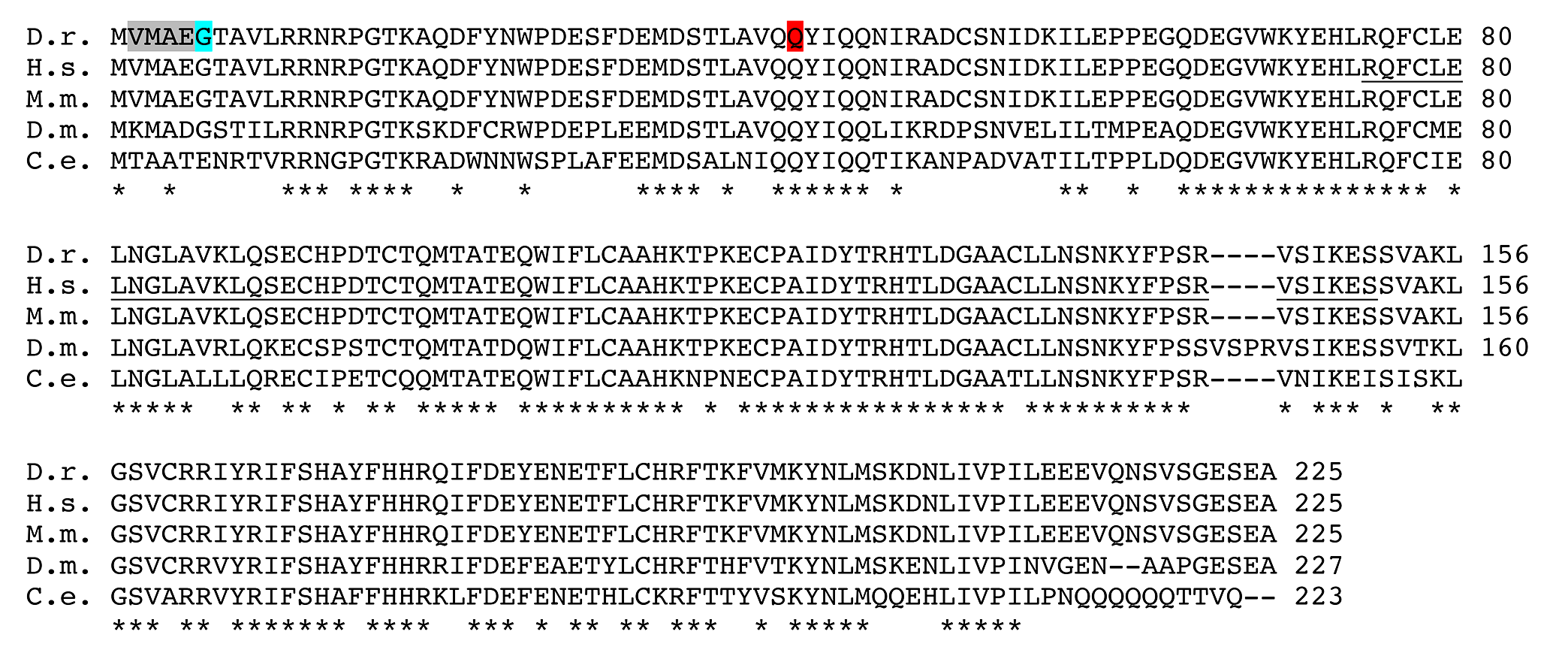

Supplement: S1 Fig — Alignment of the amino acid sequence of Mob4 from zebrafish (Danio rerio, D. r.), human (Homo sapiens, H. s.), mouse (Mus musculus, M. m.), fly (Drosophila melanogaster, D. m.) and nematode (Caenorhabditis elegans, C. e.) shows the high conservation of Mob4. Within mob4geh, the codon for glutamine at position 41 (red) was mutated into a pre-mature stop codon. In the mob4-13 allele 13 bp of coding sequence were deleted, constituting a frameshift mutation affecting the codons for the amino acids marked in grey and replacing the codon for glycine at position 6 (cyan) with a pre-mature stop codon. The peptide sequence of the immunogen of the antibodies against human MOB4 (HPA044125, Sigma) is underlined. (TIF) [file pgen.1010287.s001.tif]

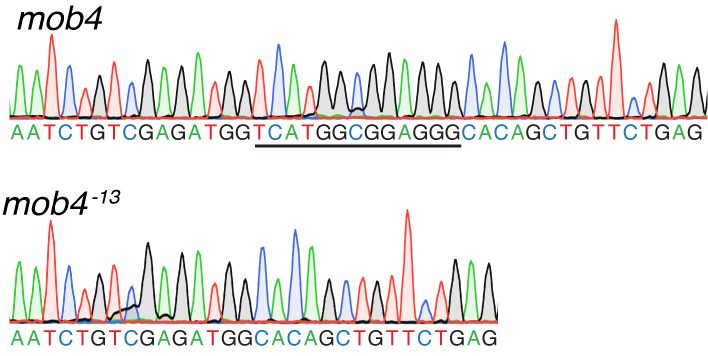

Supplement: S2 Fig — The larger context of the genomic DNA sequence of the mutation within mob4-13 shows that 13 bp (underlined) from the wildtype mob4 are deleted in mob4-13 mutants. (TIF) [file pgen.1010287.s002.tif]

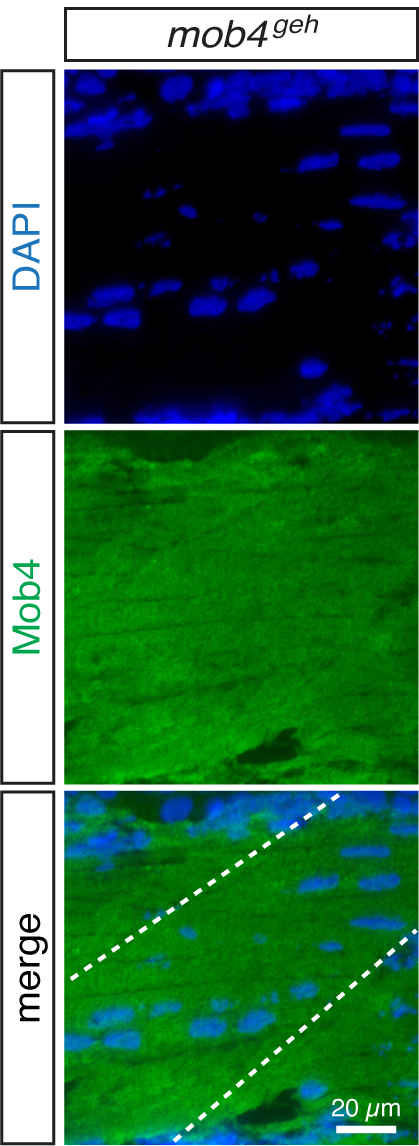

Supplement: S3 Fig — At 3 dpf, antibodies against human MOB4 (HPA044125, Sigma) did not show a specific localisation within skeletal muscle of representative mob4geh homozygotes (n = 4). Dotted lines indicate somite borders. Scale bar size is 20 μm. (TIF) [file pgen.1010287.s003.tif]

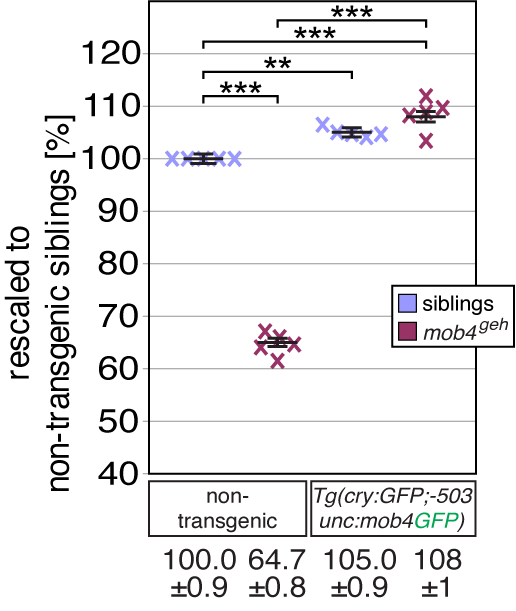

Supplement: S4 Fig — At 3 dpf, birefringence analysis revealed that directed expression of the Mob4-GFP fusion protein in the muscle via Tg(cry:GFP;-503unc:mob4-GFP) significantly rescued the birefringence reduction of non-transgenic mob4geh homozygotes. Furthermore, the birefringence of mob4geh homozygotes (108 ± 2%) and siblings (105 ± 1%) within the Tg(cry:GFP;-503unc:mob4-GFP) transgenic background was significantly higher compared to non-transgenic siblings (100 ± 1%). Crosses represent averaged birefringence of clutches with a minimum of 4 larvae per genotype (n = 5 clutches). Data are presented as mean ± SEM; *** P < 0.001 and ** P < 0.01 by one-way ANOVA with post hoc Tukey’s test. (TIF) [file pgen.1010287.s004.tif]

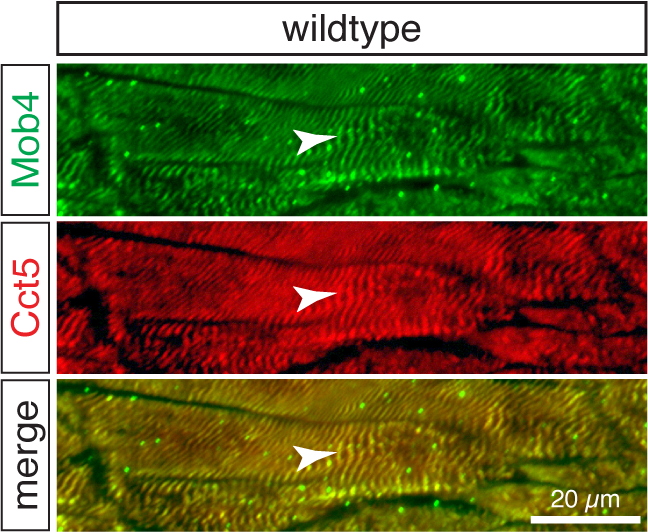

Supplement: S5 Fig — Within representative 3-dpf-old wildtype larvae, antibodies against human MOB4 (HPA044125, Sigma) co-localised with antibodies against human CCT5 (GTX110167, GeneTex) (n = 6). Scale bar size is 20 μm. (TIF) [file pgen.1010287.s005.tif]

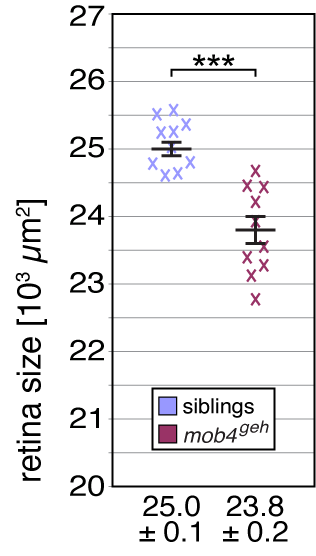

Supplement: S6 Fig — Whereas the average size of the retina of siblings was 25’000 ± 100 μm2, the retina of mob4geh homozygotes was significantly reduced to 23’800 ± 200 μm2. Crosses represent single retina sizes measured at the level of the optic nerve (n = 10). Data are presented as mean ± SEM; *** P < 0.001 by Student’s t-test. (TIF) [file pgen.1010287.s006.tif]

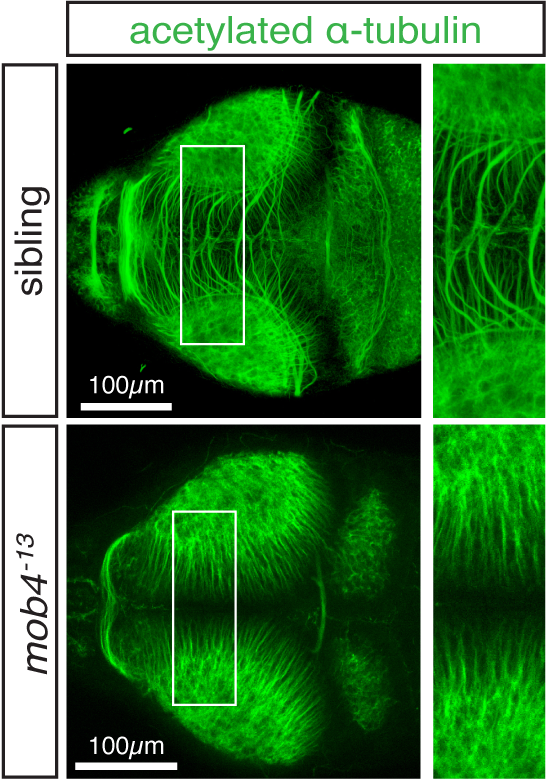

Supplement: S7 Fig — Representative Z-stacks projections of 3-dpf-old larvae immunostained with antibodies against acetylated α-tubulin documented the absence of the intertectal commissure was within mob4-13 homozygotes (n = 6 per genotype). Scale bar sizes are 100 μm. (TIF) [file pgen.1010287.s007.tif]

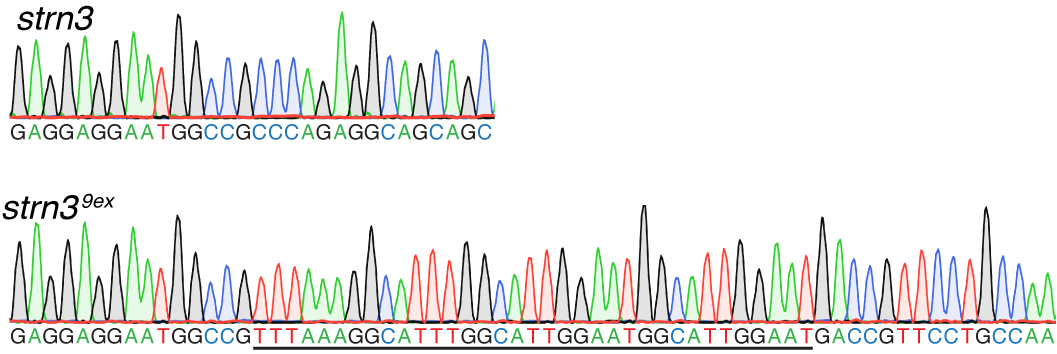

Supplement: S8 Fig — The larger context of the genomic DNA sequence of the mutation within strn39ex revealed an insertion of 35 bp (underlined) and simultaneous deletion of 23,516 bp from the wildtype strn3 allele. (TIF) [file pgen.1010287.s008.tif]
